# Prediction of colorectal cancer liver metastasis through an MRI radiomic model

**DOI:** 10.1038/s41598-026-40970-0

**Published:** 2026-02-26

**Authors:** Yao-Kun Wu, Xue Wang, Pei-Zhuo Du, Peng Zhang, Ning Liu, Yun-Yun Tao, Jing Zheng, Xiao-Hua Huang, Lin Yang

**Affiliations:** 1 Interventional Medical Center, Department of Radiology, Science and Technology Innovation Center, Affiliated Hospital of North Sichuan Medical College, Nanchong, 637000 Sichuan The People’s Republic of China; 2 Department of Radiology, Mianyang Fulin Hospital, Mianyang Fulin Hospital Co., Ltd., Mianyang, 621000 The People’s Republic of China; 3 Department of Oncology, Mianyang Fulin Hospital, Mianyang Fulin Hospital Co., Ltd., Mianyang, 621000 The People’s Republic of China; 4 Department of Radiology, Nanchong Central Hospital, Nanchong, 637000 The People’s Republic of China; 5 Department of Radiology, Hospital of the Chengdu Office of the People’s Government of the Tibetan Autonomous Region (Hospital. C.T.), Chengdu, 610041 The People’s Republic of China

**Keywords:** Radiomics, MRI, Colorectal cancer, Liver metastasis, Cancer imaging, Predictive markers

## Abstract

The aim of this study was to investigate the efficacy of a magnetic resonance imaging (MRI) radiomic model in predicting colorectal cancer liver metastasis (CRLM). Two independent cohorts consisting of 194 patients with pathologically confirmed colorectal cancer (CRC) who underwent baseline MRI examinations were recruited from the Affiliated Hospital of North Sichuan Medical College (Unit 1, n = 159) and Nanchong Central Hospital (Unit 2, n = 35) and divided into a training cohort (Unit 1) and an independent external validation cohort (Unit 2). The clinical risk factors for all patients were examined via univariate and multivariate analyses to identify independent clinical risk factors for CRLM. Radiomic features from oblique axial or axial fat-free T_2_-weighted imaging (T_2_WI) and diffusion-weighted imaging (DWI) sequences were extracted. Least absolute shrinkage and selection operator (LASSO) regression was subsequently used to screen the optimal radiomic features of each sequence. Finally, logistic regression was used to construct a prediction model according to the features from each sequence (e.g., T_2_WI and DWI models), a combined radiomic model (M) integrating the features of both the T_2_WI and DWI sequences, and a combined imaging-clinical model (U) integrating the radiomic features of both sequences with the independent clinical risk factors. The area under the receiver operating characteristic curve (AUC) was calculated to evaluate the predictive performance of each model. Among the 194 CRC patients enrolled, 86 had liver metastasis, and 108 did not. The tumor marker carcinoembryonic antigen was identified as a clinically independent risk factor for CRLM. Eleven optimal radiomic features were screened from each of the T_2_WI and DWI sequences through LASSO regression analysis. The AUC values of the clinical, T_2_WI, DWI, M, and U models were 0.755, 0.834, 0.844, 0.853, and 0.890, respectively, in the training cohort and 0.697, 0.786, 0.750, 0.808, and 0.842, respectively, in the validation cohort. The predictive performance of the combined models was better than that of the single-sequence models, and the U model performed best in terms of predicting CRLM. The results of this study suggest that the MRI radiomic model based on the imaging features of primary CRC lesions integrated with clinical risk factors can accurately predict CRLM, which may assist clinicians in the development of individualized treatment plans for patients with CRC.

## Introduction

Colorectal cancer (CRC) is the third most commonly diagnosed malignancy and the second leading cause of cancer-related death worldwide^1,2^. Approximately 15–25% of CRC patients already present with liver metastasis (LM) upon diagnosis, and 15–25% of CRC patients who receive radical surgical treatment will also develop LM^3–7^. Studies have shown that LM is the leading cause of death in CRC patients, and the median survival of patients with untreated colorectal liver metastasis (CRLM) is only 6.9 months; furthermore, the 5-year survival rate is < 5%^8–10^. However, for patients who undergo radical resection for LM or who demonstrate no evidence of disease (NED), the median survival time is 35 months, and the 5-year survival rate is 30–57%^9,10^. Early detection and active individualized treatment of LM are very important for improving the prognosis of CRC patients. Traditional imaging methods have been widely used in the preoperative clinical assessment of CRLM; however, their diagnostic accuracy remains unsatisfactory^11,12^. Although some clinicopathological features can be used to assess the potential risk of CRLM, these indicators can be obtained only after radical resection^13,14^. Therefore, there is an urgent clinical need for noninvasive and accurate preoperative predictors of CRLM.

The concept of radiomics was first proposed by Lambin et al. in 2012^15^. Radiomics has since been gradually used to achieve disease classification and prognosis prediction^16–21^. Currently, magnetic resonance imaging (MRI) is the preferred method for the clinical assessment of CRLM; among the various MRI modalities, T_2_-weighted imaging (T_2_WI) and diffusion-weighted imaging (DWI) sequences obtained via axial MRI are key for finely annotating CRC. Most existing studies on the radiomic prediction of CRLM are based on imaging data from the liver parenchyma^22–25^; a few studies have used radiomic models based on baseline MRI examinations of the primary lesion in CRC patients to predict CRLM. Liang et al.^26^ constructed a radiomic prediction model based on MRI T_2_WI and venous-phase images of the primary lesion in the RC. The results showed that the baseline MRI-omics model based on primary lesion images had predictive potential for LM. Liu et al.^27^ established a prediction model using radiomics signatures of the T_2_W images of 127 primary RC patients combined with the levels of the tumor markers CEA and CA19-9, which showed good performance in predicting LM. However, none of these studies investigated the role of fine-annotated key sequences (such as DWI) in CRC. Recently, Li et al.^28^ reported the effectiveness of radiomics, which is based on multiparametric MRI examinations of first-diagnosed rectal cancer patients, in predicting rectal cancer-related LM. The results revealed that the AUC value of the DWI + HD T_2_WI joint model in the testing set was significantly greater than that of the DWI or HD T2 model alone. The integration of radiomic features into the clinical model improved the predictive performance. However, very few studies have investigated the ability of MR T_2_WI and DWI-based radiomic models to predict CRLM. The aim of this study was to investigate the value of a radiomic model based on baseline MR T_2_W1 and DWI sequences of CRC primary lesions in predicting CRLM. Additionally, Shapley additive explanations (SHAP) interpretability analysis was employed to quantify the degree of contribution of each feature in the optimal model.

## Materials and methods

### Patients

**Fig. 1:**
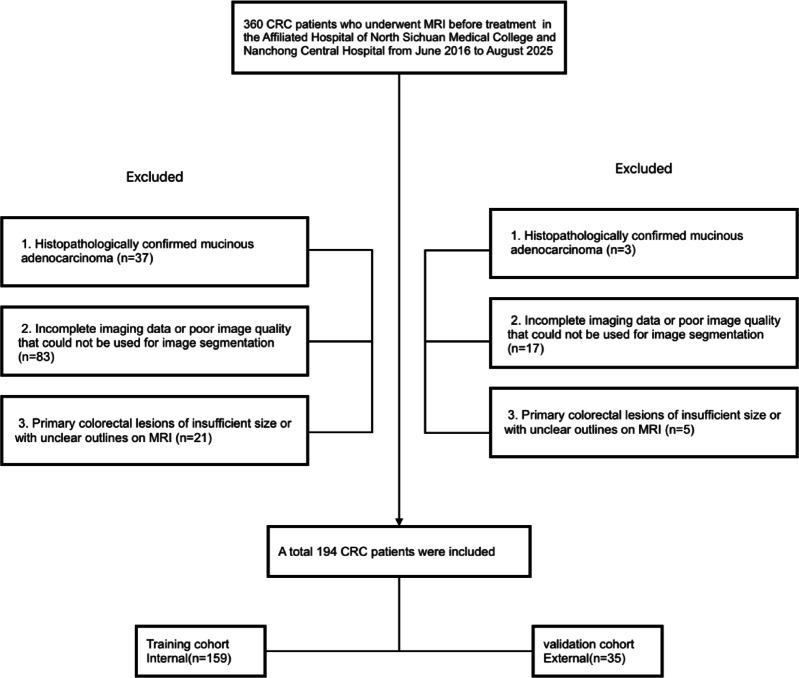
Flowchart of patient enrollment.

This study was approved by the Ethics Committee of the Affiliated Hospital of North Sichuan Medical University (No. 2023ER77-1) and was conducted in accordance with the Declaration of Helsinki. All methods were performed in accordance with the relevant guidelines and regulations, and written informed consent was obtained from all participants. The study subjects included CRC patients who underwent MRI examinations at the Affiliated Hospital of North Sichuan Medical College (Unit 1) and Nanchong City Central Hospital (Unit 2). The patient inclusion criteria were as follows: (1) CRC confirmed by colonoscopic biopsy or postoperative histopathology; (2) no other malignant tumors; (3) complete baseline MR images of good image quality prior to treatment and no previous antitumor treatment prior to the baseline MR examination; and (4) no LMs at baseline but LMs present at the 1-year follow-up. The exclusion criteria were as follows: (1) histopathologically confirmed mucinous adenocarcinoma (because of the high risk of developing LM and poor prognosis)^26^; (2) incomplete imaging data or image quality insufficient for segmentation; and (3) primary colorectal lesions of insufficient size or with unclear outlines on MRI. This group of LM patients was diagnosed by liver biopsy, pathological examination of surgical resection, and enhanced CT/MRI or ultrasound examination for typical metastasis features. A total of 360 patients were initially screened at the two units; of these, 159 patients from Unit 1 met the inclusion criteria and were placed in the training cohort, while 35 eligible CRC patients from Unit 2 were included in the external validation cohort, resulting in a total of 194 patients (Fig. 1).

The following clinical data were collected from the patients: sex, age, MRI T stage, MRI N stage, CEA level and CA19-9 level. MRI TN staging was performed according to the 8th edition of the TNM staging system of the American Cancer Society^29^. The interval between the collection of blood samples for the detection of preoperative CEA and CA19-9 levels and the baseline MRI examination was not more than 2 weeks. The clinical data of the patients were analyzed via univariate and multivariate Cox regression to identify independent predictors of CRLM.

### MRI acquisition

In this study, a standardized rectangular cancer MRI scan protocol was employed with a Discovery 750 3.0 T superconducting MRI scanner with 32-channel phased-array surface coils (GE Medical Systems LLC, 3200 N. Grandview Blvd., Waukesha, WI 53118, USA) (Unit 1) and a United Imaging uMR 588 1.5 T MRI scanner with 6-channel phased-array surface coils (United Imaging Healthcare, Shanghai, China) (Unit 2). All patients fasted for 4 h before examination, and a glycerol enema (20 mL of glycerol) was used to cleanse the intestinal tract before examination. Anisodamine or scopolamine (20 mg) was intramuscularly injected half an hour before the examination (none of the patients presented with contraindications before the injection) to prevent movement artifacts caused by physiological peristalsis of the gastrointestinal tract, bladder or other organs. The acquisition sequences included standard sagittal or coronal T_2_W images without fat compression, (oblique) axial T_2_W images without fat compression, and high b-value (b = 800) DW images (where the oblique axial view refers to the body position perpendicular to the long axis of the rectal lesion). Standard sagittal or coronal T_2_W images without fat compression were used to determine the location and boundary of the lesion, and the (oblique) axial T_2_W and DW images without fat compression were used as region of interest (ROI) annotation sequences (Table 1).

**Table 1:** Axial T_2_WI and DWI parameters for colorectal cancer.

Sequence	TR/TE (ms)	ST (mm)	Matrix (mm^2^)	FOV (mm^2^)	FA (°)
Axial T_2_WI	1700–5050/110–120	3–6	320–384 × 256	200–360 × 200–360	90
Axial DWI	3000–7000/50–80	4–6	128–160 × 192	340–380 × 340–380	90

TR, repetition time; TE, echo time; ST, section thickness; FOV, field of view; FA, flip angle.

### Image segmentation and feature extraction

**Fig. 2:**
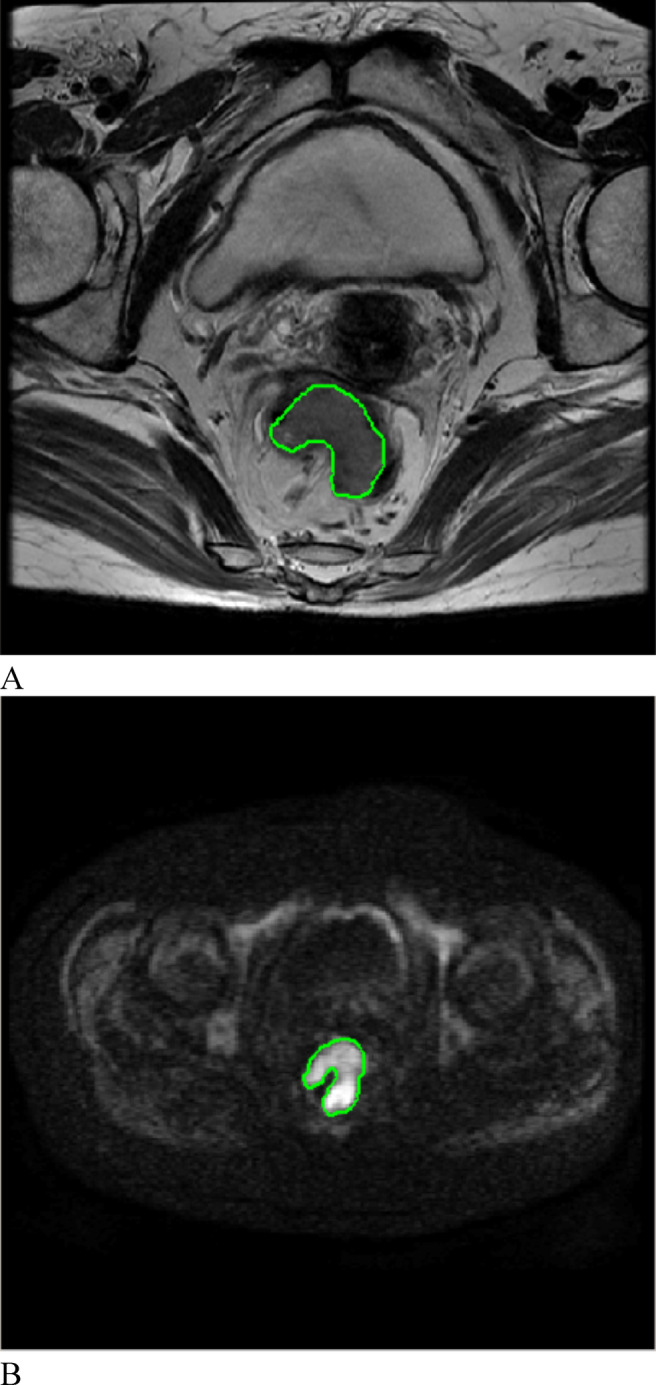
On oblique axial T_2_WI and DWI, the ROI was manually delineated layer by layer along the edge of the rectal cancer lesion. (**A**) T_2_-weighted imaging; (**B**) diffusion-weighted imaging.

All MR images of the 194 patients with CRC were retrieved in DICOM format from the Picture Archiving and Communication Systems (PACSs). To minimize the impact of different MRI scanners and acquisition parameters on the radiomic analysis, all the images were preprocessed before feature extraction. Preprocessing included resampling to an isotropic voxel size of 3.0 × 3.0 × 3.0 mm^3^, gray-level discretization with a fixed bin width of 25, and gray-level normalization to standardize the voxel intensities across patients^30,31^. The preprocessed MR images were subsequently imported into IBEX software (β1.0, http://bit.ly/IBEXMDAnderson) in DICOM format for segmentation of the tumor lesions. Radiologist A (Yao-Kun Wu), with 2 years of work experience, delineated each lesion layer by layer on (oblique) axial T_2_W and DW images to generate a volume of interest (VOI) (Fig. 2). Gas in the intestinal lumen, cystic degeneration between the lesion and the normal bowel, necrotic and transitional areas and adipose tissue around the intestinal wall were avoided. The operator was not aware of the basic information of the patient, such as the clinical and pathological results, before the target volume was delineated. Finally, the radiomic features were extracted, and the T_2_WI and DWI feature datasets were generated.

### Feature screening

The intraobserver and interobserver consistency of the features were evaluated by determining the intraclass correlation coefficients (ICCs). For the intraobserver consistency test, radiologist A randomly selected 1/3 of the patients from the entire cohort to repeat ROI delineation within 1 week. For the interobserver consistency test, the MR images of selected cases were independently segmented by radiologist A and radiologist B (Ning Liu, with 4 years of work experience). Features with ICCs < 0.75 were excluded. A feature with an ICC ≥ 0.75 was considered to have good consistency, and this feature was retained and entered the next step of screening.

Next, the maximum relevance minimum redundancy (mRMR) algorithm was applied to the training cohort to eliminate redundant and irrelevant features. The least absolute shrinkage and selection operator (LASSO) algorithm with fivefold cross-validation was subsequently employed to determine the optimal hyperparameters and further refine the selected features.

### Model construction and evaluation

After radiomics feature extraction and screening, logistic regression analysis was performed to identify the optimal radiomic features and to construct the radiomic models. Receiver operating characteristic (ROC) curves were generated, and the area under the curve (AUC) was calculated to evaluate the predictive performance of the radiomic and combined models. Model accuracy, sensitivity, specificity, positive predictive value (PPV), and negative predictive value (NPV) in predicting colorectal cancer liver metastasis were assessed, and validation was conducted in the independent cohort.

### Statistical methods

R statistical software (4.2.2, https://www.r-project.org/) was used for statistical analysis. For continuous variables, the *Shapiro–Wilk* test was used to determine normality, and the *Bartlett* test was used to determine the homogeneity of variance. Data satisfying the conditions of normality and homogeneity of variance were analyzed with the independent sample *t* test; otherwise, the *Mann–Whitney U* test was used, and in both cases, the mean value was used to describe the data. Categorical variables were analyzed using the chi-square test and are presented as percentiles. A two-sided *P* value < 0.05 was considered to indicate statistical significance. Calibration curves were plotted, and the Hosmer–Lemeshow test was performed to determine the goodness-of-fit of the calibration. Decision curve analysis (DCA) was performed to quantify the net clinical benefits of the models across a range of threshold probabilities. Additionally, SHAP interpretability analysis was employed to quantify the degree of contribution of each feature in the optimal model.

## Results

### Patient characteristics

A total of 194 patients with pathologically confirmed CRC were included; 159 from the Affiliated Hospital of North Sichuan Medical College (training cohort) and 35 from Nanchong Central Hospital (validation cohort). Among them, 76 patients in the training cohort and 10 patients in the validation cohort had liver metastasis, whereas 83 and 25 patients, respectively, did not. In the training cohort, patients with CRLM (+) were significantly older than those with CRLM (−) were (64.05 ± 11.80 vs. 58.98 ± 12.73 years, P = 0.010). Serum levels of tumor markers, including CEA (310.77 ± 1315.32 vs. 4.99 ± 6.81 ng/mL, P < 0.001) and CA19-9 (1549.28 ± 9407.46 vs. 17.32 ± 14.10 U/mL, P < 0.001), were markedly elevated in patients with CRLM. No significant differences in sex distribution (P = 0.132), MRI T stage (P = 0.988), or MRI N stage (P = 0.887) were detected between the two groups. In the validation cohort, no significant differences were found in age (65.90 ± 10.58 vs. 61.60 ± 11.10 years, P = 0.302), sex (male: 70.0% vs. 68.0%, P = 1.000), MRI T stage (P = 0.302), or MRI N stage (P = 0.439) between the CRLM (+) and CRLM (−) patients. CEA levels remained significantly greater in the CRLM ( +) group (108.10 ± 313.64 vs. 6.10 ± 13.28 ng/mL, P = 0.050) than in the CRLM (−) group, whereas CA19-9 levels were not significantly different (19.64 ± 11.42 vs. 16.70 ± 25.59 U/mL, P = 0.083) between the two groups, likely reflecting the limited sample size and substantial interindividual variability (Table 2).

**Table 2:** Clinical features of patients with CRLM( +) and CRLM(−) in the training and validation cohorts.

	Training			Validation		
	CRLM(+) (*n* = 76)	CRLM(-) (*n* = 83)	*P* value	CRLM(+) (*n* = 10)	CRLM(-) (*n* = 25)	*P* value
Age/yr (Mean ± SD)	64.05 ± 11.80	58.98 ± 12.73	0.010	65.90 ± 10.58	61.60 ± 11.10	0.302
Sex (%)			0.132			1.000
Male	57(75.0)	52(62.7)		7(70.0)	17(68.0)	
Female	19(25.0)	31(37.3)		3(30.0)	8(32.0)	
MRT stage (%)			0.988			0.302
T1	0(0)	1(1.2)		0(0)	1(4.0)	
T2	1(1.3)	14(16.9)		1(10.0)	2(8.0)	
T3	48(63.2)	46(55.4)		3(30.0)	15(60.0)	
T4	27(35.5)	22(26.5)		6(60.0)	7(28.0)	
MRN stage (%)			0.887			0.439
N0	13(17.1)	20(24.1)		1(10.0)	7(28.0)	
N1	22(28.9)	22(26.5)		3(30.0)	8(32.0)	
N2	41(53.9)	41(49.4)		6(60.0)	10(40.0)	
CEA level (ng/mL)	310.77 ± 1315.32	4.99 ± 6.81	< 0.001	108.10 ± 313.64	6.10 ± 13.28	0.050
CA19-9 level (U/mL)	1549.28 ± 9407.46	17.32 ± 14.10	< 0.001	19.64 ± 11.42	16.70 ± 25.59	0.083

CRLM, colorectal cancer liver metastasis; CEA, carcinoembryonic antigen; CA19-9, carbohydrate antigen 19–9.

### Feature extraction and selection

**Fig. 3:**
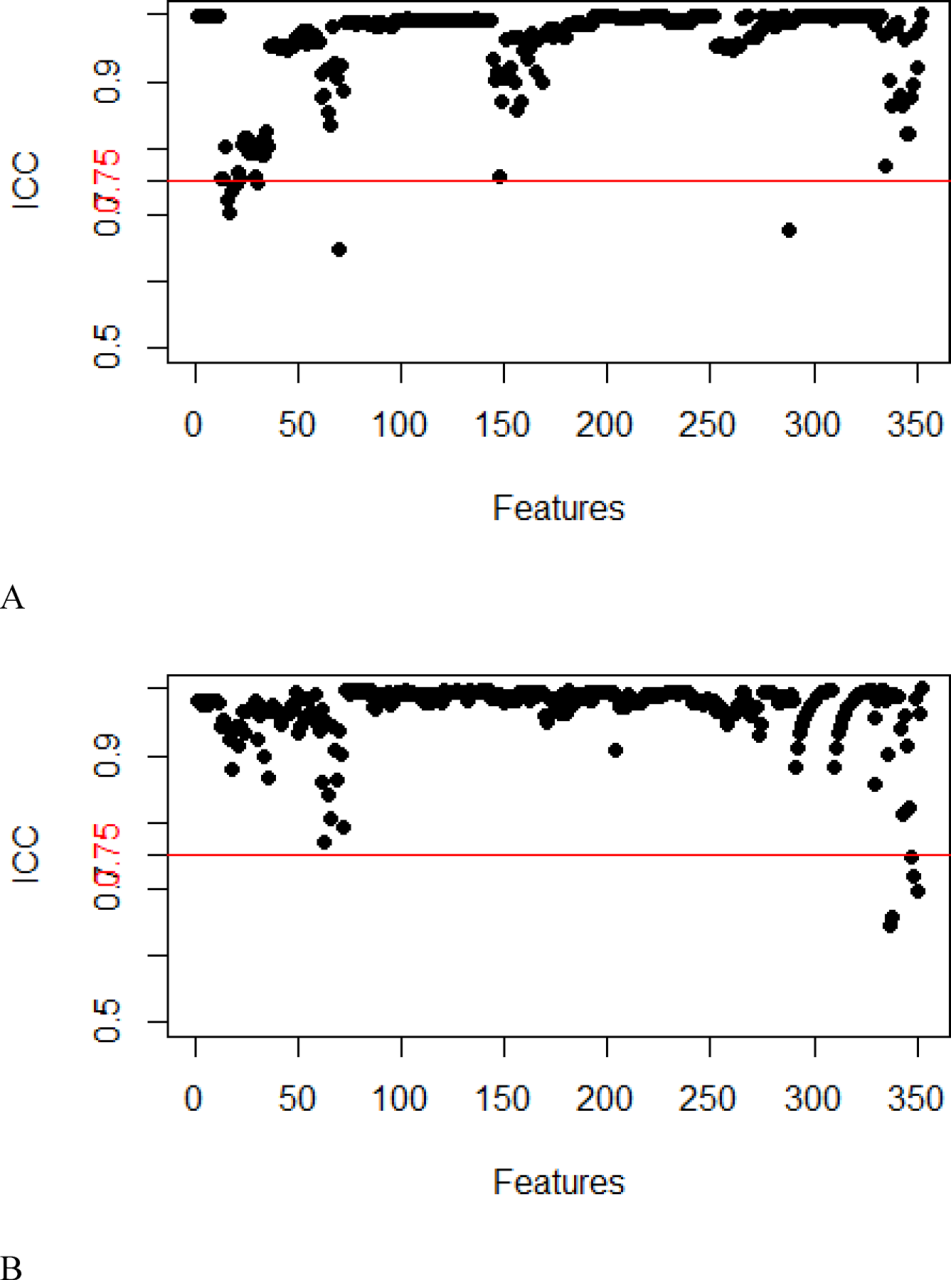
Intragroup consistency test: evaluation of the reproducibility of MRI radiomic feature extraction by the intraclass correlation coefficient (ICC). (**A**) T_2_-weighted imaging features; (**B**) diffusion-weighted imaging features.

**Fig. 4:**
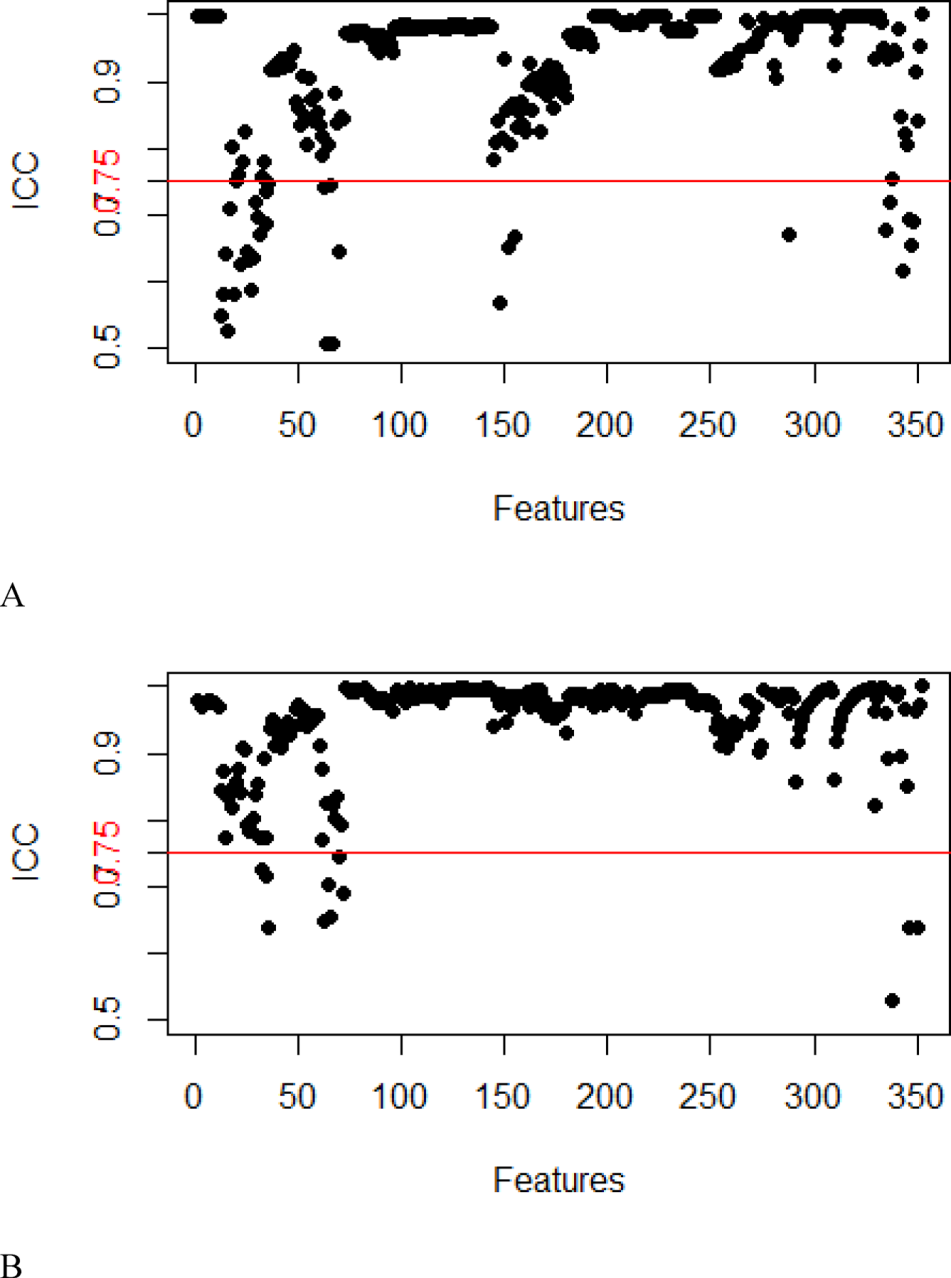
Intergroup consistency test: evaluation of the repeatability of MRI radiomic feature extraction by the intraclass correlation coefficient (ICC). (**A**) T_2_-weighted imaging; (**B**) Diffusion-weighted imaging.

**Fig. 5:**
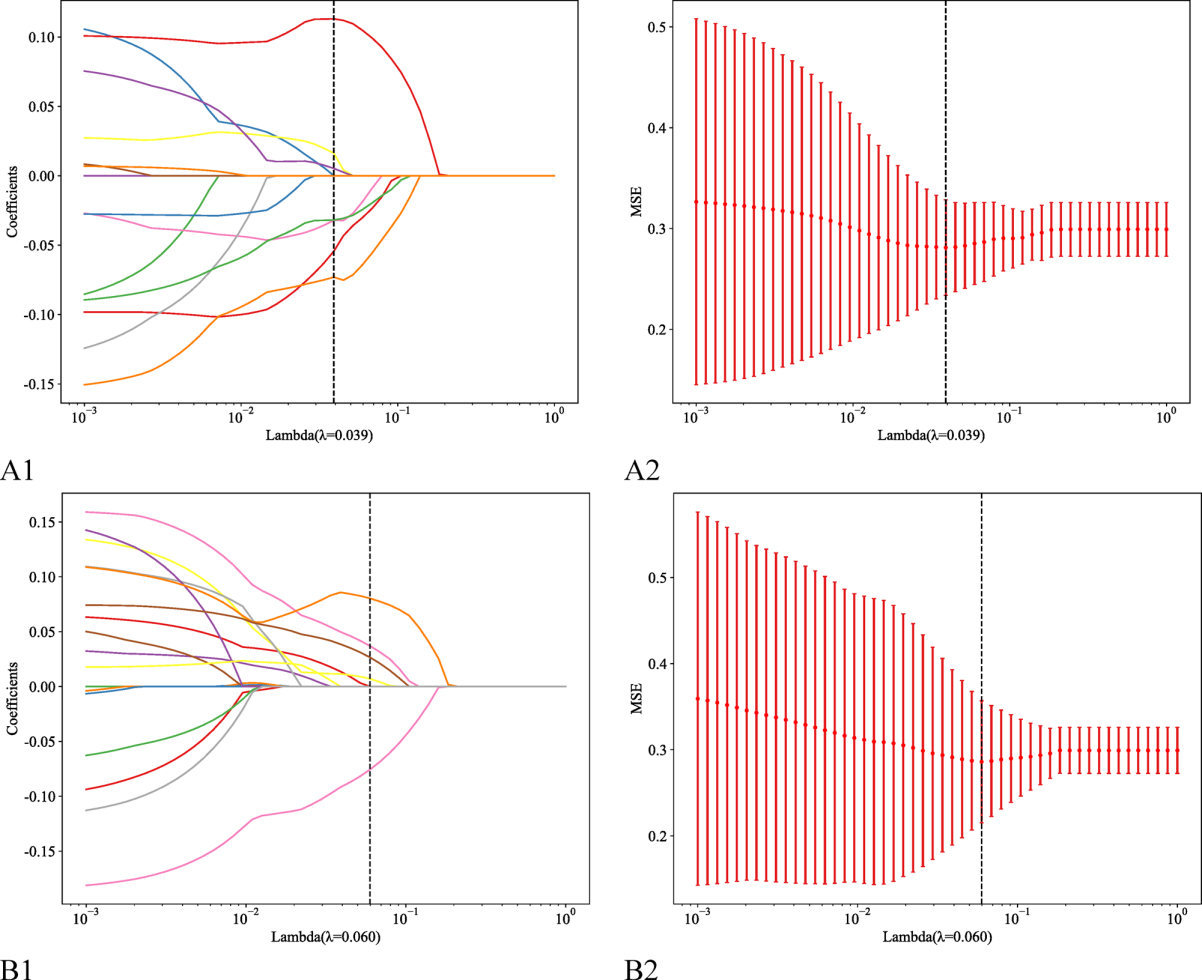
Feature selection using least absolute shrinkage and selection operator (LASSO) regression to predict CRLM. (**A1**-**A2**) T_2_-weighted imaging. (**B1**-**B2**) Diffusion-weighted imaging.

In this study, a total of 352 features were extracted from the T_2_W and DW images. After consistency analysis (Figs. 3 and 4), univariate analysis and least absolute shrinkage and selection operator (LASSO) analysis (Fig. 5) for dimensionality reduction revealed 5 and 6 optimal features, respectively (Table 3)..

**Table 3:** Selected optimal features for CRLM prediction.

Cohort	Feature type	Feature name
T_2_WI	GLCM	135-1Contrast
	Shape	90-4Correlation 135-7Energy Max3D Diameter VoxelSize
DWI	GLCM	135-4ClusterShade 135-1InformationMeasureCorr1 135-7InverseVariance
	Shape	Max3D Diameter NumberOfVoxel SurfaceAreaDensity

CRLM, colorectal cancer liver metastasis; GLCM, gray-level co-occurrence matrix.

### Model evaluation

**Fig. 6:**
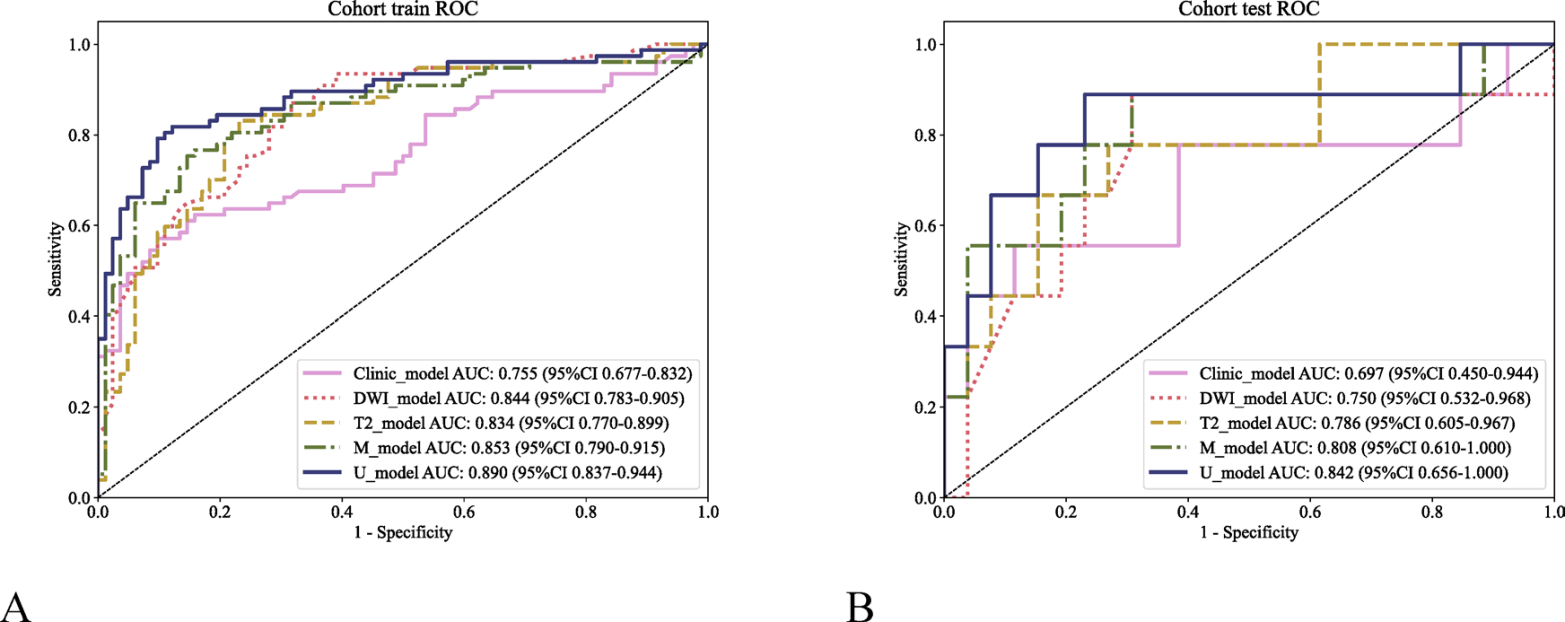
Receiver operating characteristic (ROC) curves showing the predictive performance of the clinical model, T_2_WI model, the DWI model, the M model, and the U model for CRLM in the training (**A**) and validation (**B**) groups.

The AUC values of the clinical, T_2_WI, DWI, M, and U models were 0.755, 0.834, 0.844, 0.853, and 0.890, respectively, in the training cohort and 0.697, 0.786, 0.750, 0.808, and 0.842, respectively, in the validation cohort. Among them, the combined clinical-radiomics model (the U model) performed the best in predicting CRLM (Table 4, Fig. 6).

**Table 4:** Performance of the constructed models in predicting CRLM.

	Model	AUC	Sensitivity	Specificity	PPV	NPV	Accuracy	F1
	Clinical_model	0.755	0.571	0.902	0.846	0.692	0.742	0.682
Training cohort	DWI_model	0.844	0.870	0.683	0.720	0.848	0.774	0.788
	T2_model	0.834	0.831	0.768	0.771	0.829	0.799	0.800
	M_model	0.853	0.766	0.841	0.819	0.793	0.805	0.792
	U_model	0.890	0.818	0.878	0.863	0.837	0.849	0.840
	Clinical_model	0.697	0.556	0.885	0.625	0.852	0.800	0.588
Validation cohort	DWI_model	0.750	0.889	0.692	0.500	0.947	0.743	0.640
	T2_model	0.786	0.667	0.846	0.600	0.880	0.800	0.632
	M_model	0.808	0.889	0.692	0.500	0.947	0.743	0.640
	U_model	0.842	0.889	0.769	0.571	0.952	0.800	0.696

CRLM, colorectal cancer liver metastasis; M model, multisequence radiomic model; U model, union of the multisequence radiomic model and clinical model; AUC, area under the receiver operating characteristic curve; Sen, sensitivity; Spe, specificity; PPV, positive predictive value; NPV, negative predictive value; and ACC, accuracy.

**Fig. 7:**
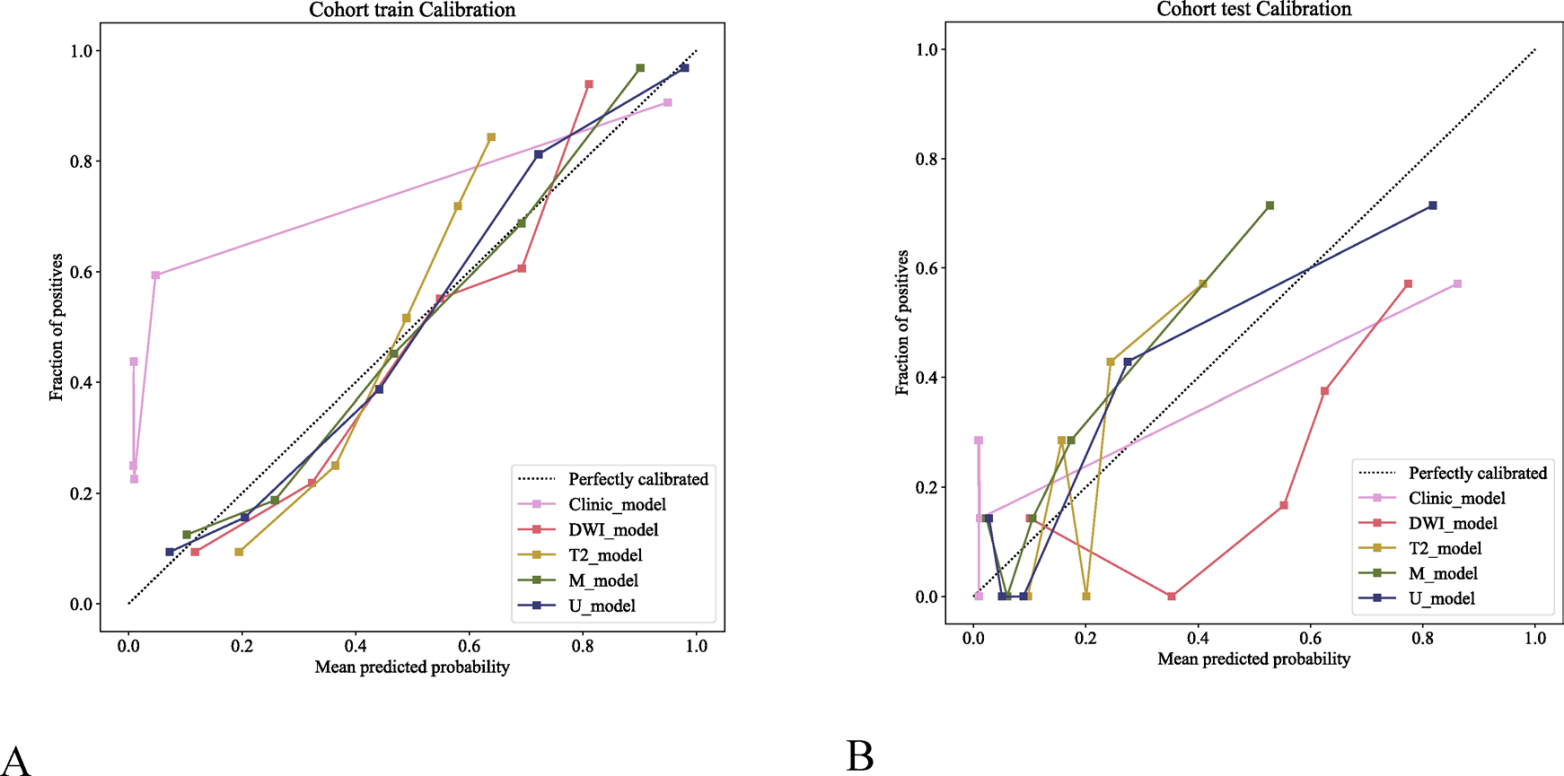
Calibration curves of the different models (Clinical_model, DWI_model, T2_model, M_model, U_model) in the training (**A**) and external validation cohort (**B**) cohorts demonstrate that both the single radiomic models and the combined models exhibited good agreement between the predicted and actual outcomes in the training and validation cohorts, with the U model showing superior performance. The x-axis represents the mean predicted probability, and the y-axis represents the observed proportion of positives. The dashed line indicates the ideal calibration (perfectly calibrated).

**Fig. 8:**
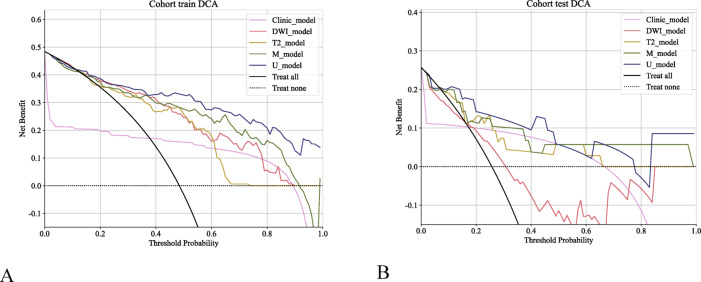
Decision curve analysis (DCA) of the different models revealed that compared with the clinical and single radiomic models, the U model consistently achieved greater net clinical benefits in both the training and validation cohorts. (**A**) DCA curves of the clinical_model, DWI_model, T2_model, M_model, and U_model in the training cohort. (**B**) DCA curves of the models in the external validation cohort. The x-axis represents the threshold probability, and the y-axis represents the net clinical benefits.

The calibration curves demonstrate that both the single radiomic models and the combined models exhibited good agreement between the predicted and actual outcomes in the training and validation cohorts, with the U model showing superior performance (Fig. 7). The decision curve analysis plots revealed that, in both the training and validation cohorts, the U model consistently achieved greater net clinical benefits than the clinical and single radiomic models did (Fig. 8).

### SHAP interpretability analysis

**Fig. 9:**
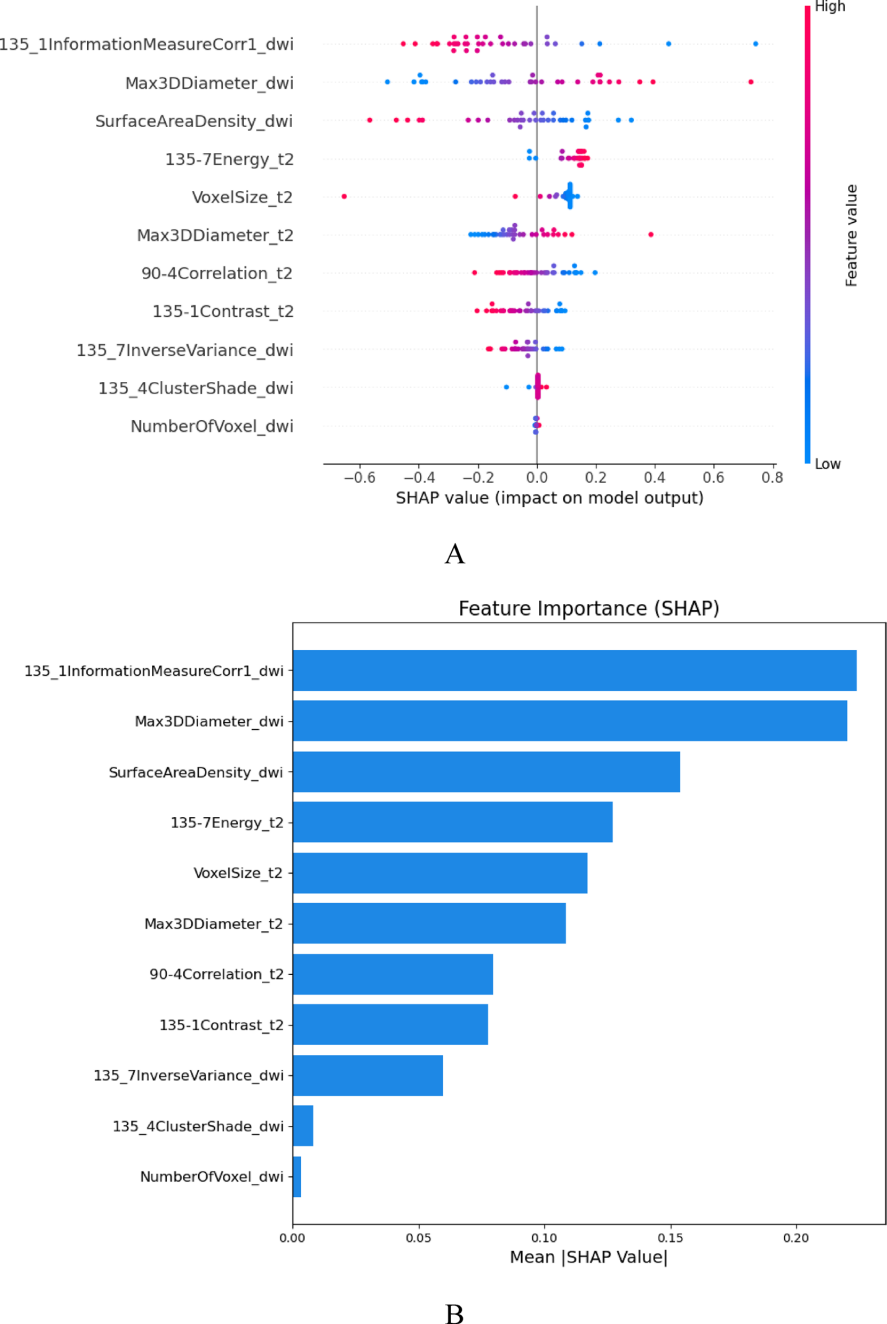
SHAP analysis plot of each predictor in the radiomic model. SHAP summary plot (**A**) shows the distribution of the SHAP values of each predictor in all the samples. The SHAP bar plot (**B**) shows the importance of each feature included in the model. The results of the joint radiomics model revealed that 135_1InformationMeasureCorr1_dwi had the greatest contribution.

To further analyze the importance of the features included in the model, SHAP interpretability analysis was used to quantify the contributions of these features (Fig. 9). The results revealed that “135_1InformationMeasureCorr1_dwi” had the strongest influence on the final prediction result in the M model.

## Discussion

In this study, on the basis of the preoperative baseline T_2_WI and DWI radiomic characteristics of the primary CRC lesion, we created a T_2_WI model, a DWI model and a T_2_W + DWI model (M model) and integrated all the optimal imaging characteristics and clinical risk factors to construct a clinical–radiomic joint model (U model) to predict CRLM. The results showed that the predictive performance of the T_2_WI + DWI joint model was better than that of the T_2_WI or DWI model alone, while the joint clinical-radiomics model had the best predictive performance. The DCA curves revealed an enhanced clinical net benefit from the joint clinical-radiomics model, while the calibration curves confirmed its good predictive consistency. In addition, our methodology introduces SHAP analysis to interpret the optimal model. Combined with SHAP analysis, the contribution of each feature in this model is explained, which helps clinicians better understand the predictive model and develop personalized treatment plans. The results of the present study are consistent with those reported by Li et al.^28^.

In our study, shape and GLCM features were identified as significant discriminators between CRLM and non-CRLM patients. Shape features quantify the geometric properties of the region of interest, such as diameter, surface area, and contour irregularity. An increased mean tumor diameter has been shown to be associated with a greater likelihood of metastatic disease^32^. Moreover, GLCM features, which can be used to assess intratumoral heterogeneity by reflecting spatial gray-level dependencies, have demonstrated significant correlations with aggressive tumor phenotypes and suboptimal treatment responses^33^. Tumor heterogeneity is widely recognized as a characteristic of malignancy associated with adverse tumor biology^34^. Joung JG et al. reported that tumor heterogeneity across CRC samples was highly variable; a high degree of tumor heterogeneity was associated with poor disease-free survival (DFS), and highly heterogeneous primary CRC was correlated with a high rate of LM^35^. In recent years, studies have revealed that radiomics can characterize tumor heterogeneity, which is helpful for clinical decision-making^36–38^. In the present study, the joint radiomics model based on the two CRC key annotation sequences, T2WI and DWI, achieved ideal performance. The results of the SHAP analysis of the radiomics joint model revealed that 135_1InformationMeasureCorr1_dwi (texture feature) had the highest contribution value, suggesting that texture features may be more strongly related to tumor heterogeneity, which is consistent with the results of a previous study^39^.

Additionally, our univariable comparisons revealed that serum CEA levels were an independent clinical predictor of CRLM, whereas CA19-9 levels were significantly associated with CRLM but did not significantly differ after multivariable adjustment. These findings are in line with reports by Zhu et al.^40^ and Zhang et al.^41^. Li et al.^42–44^ reported that TNM stage was a risk factor for CRLM. However, in the present study, there was no difference in the T or N stage of CRC patients between the liver metastasis group and the group without liver metastasis, which may be related to the small sample size of this study.

This study has the following limitations. (1) The sample size of the present study was relatively small, which may have affected the reliability of the model. In the future, studies with larger sample sizes should be conducted for further validation to strengthen the findings and improve the applicability of the results^45^. (2) As this was a retrospective study, it may have been affected by biases inherent to this study design. For example, several important pathological features, such as the degree of tumor differentiation and microvascular invasion (MVI) status, were not included. This study could benefit from a more extensive exploration of other clinical variables that may influence metastasis risk. Future prospective studies should be conducted to strengthen the validity of the findings of the present study. Additionally, clinical applications should be further studied.

## Conclusions

The radiomic model based on features from the T_2_WI and DWI sequences of CRC primary lesions could predict CRLM well; the combination of independent clinical risk factors and radiomic features further improved the predictive performance of the model. The results of the present study may assist clinicians in developing individualized treatment plans for patients with CRC.

## Data Availability

The data are provided within the manuscript or supplementary information files.
